# The role of *CSNK1A1* and its de novo mutations in infantile spasms syndrome

**DOI:** 10.1093/hmg/ddaf030

**Published:** 2025-03-28

**Authors:** Decheng Ren, Zhenxi Yang, Juan Hu, Lei Ji, Yan Bi, Fan Yuan, Yang Yan, Jing Peng, Keyi Li, Ke Yang, Liangjie Liu, Xiao Mao, Yingying Luo, Yanlin Wang, Guang He, Kai Li, Ying Peng

**Affiliations:** Bio-X Institutes, Key Laboratory for the Genetics of Developmental and Neuropsychiatric Disorders, Shanghai Jiao Tong University, No. 800, Dongchuan Road, Minhang District, Shanghai, 201109, China; Bio-X Institutes, Key Laboratory for the Genetics of Developmental and Neuropsychiatric Disorders, Shanghai Jiao Tong University, No. 800, Dongchuan Road, Minhang District, Shanghai, 201109, China; Rehabilitation medicine department, Wuhan Children's Hospital, Tongji Medical College, Huazhong University of Science & Technology, No. 100, Xianggang Road, Jiang'an District, Wuhan, Hubei province, 430016, China; Bio-X Institutes, Key Laboratory for the Genetics of Developmental and Neuropsychiatric Disorders, Shanghai Jiao Tong University, No. 800, Dongchuan Road, Minhang District, Shanghai, 201109, China; Department of Prenatal Diagnosis Center, International Peace Maternity and Child Health Hospital of China Welfare Institute, No. 1961, Huashan Road, Xuhui District, Shanghai, 200030, China; Bio-X Institutes, Key Laboratory for the Genetics of Developmental and Neuropsychiatric Disorders, Shanghai Jiao Tong University, No. 800, Dongchuan Road, Minhang District, Shanghai, 201109, China; Bio-X Institutes, Key Laboratory for the Genetics of Developmental and Neuropsychiatric Disorders, Shanghai Jiao Tong University, No. 800, Dongchuan Road, Minhang District, Shanghai, 201109, China; Bio-X Institutes, Key Laboratory for the Genetics of Developmental and Neuropsychiatric Disorders, Shanghai Jiao Tong University, No. 800, Dongchuan Road, Minhang District, Shanghai, 201109, China; Bio-X Institutes, Key Laboratory for the Genetics of Developmental and Neuropsychiatric Disorders, Shanghai Jiao Tong University, No. 800, Dongchuan Road, Minhang District, Shanghai, 201109, China; Bio-X Institutes, Key Laboratory for the Genetics of Developmental and Neuropsychiatric Disorders, Shanghai Jiao Tong University, No. 800, Dongchuan Road, Minhang District, Shanghai, 201109, China; Bio-X Institutes, Key Laboratory for the Genetics of Developmental and Neuropsychiatric Disorders, Shanghai Jiao Tong University, No. 800, Dongchuan Road, Minhang District, Shanghai, 201109, China; Prenatal Diagnosis Center, National Health Commission Key Laboratory of Birth Defects for Research and Prevention, Hunan Provincial Maternal and Child Health Care Hospital, No. 53, Xiangchun Road, Kaifu District, Changsha, Hunan province, 410008, China; Bio-X Institutes, Key Laboratory for the Genetics of Developmental and Neuropsychiatric Disorders, Shanghai Jiao Tong University, No. 800, Dongchuan Road, Minhang District, Shanghai, 201109, China; Department of Prenatal Diagnosis Center, International Peace Maternity and Child Health Hospital of China Welfare Institute, No. 1961, Huashan Road, Xuhui District, Shanghai, 200030, China; Bio-X Institutes, Key Laboratory for the Genetics of Developmental and Neuropsychiatric Disorders, Shanghai Jiao Tong University, No. 800, Dongchuan Road, Minhang District, Shanghai, 201109, China; Prenatal Diagnosis Center, National Health Commission Key Laboratory of Birth Defects for Research and Prevention, Hunan Provincial Maternal and Child Health Care Hospital, No. 53, Xiangchun Road, Kaifu District, Changsha, Hunan province, 410008, China; Department of Neurology and Suzhou Clinical Research Center of Neurological Disease, The Second Affiliated Hospital of Soochow University, No. 1055, Sanxiang Road, Gusu District, Suzhou, Jiangsu province, 215004, China; Prenatal Diagnosis Center, National Health Commission Key Laboratory of Birth Defects for Research and Prevention, Hunan Provincial Maternal and Child Health Care Hospital, No. 53, Xiangchun Road, Kaifu District, Changsha, Hunan province, 410008, China

**Keywords:** Infantile spasms syndrome, CSNK1A1, β-catenin, cell proliferation

## Abstract

Infantile spasms syndrome (ISS) is an early-onset epileptic encephalopathy characterized by uncontrollable seizures, severe electroencephalogram abnormalities, as well as delayed cognitive and behavioral development. Independent studies have shown that a variety of genes are involved in ISS and genetic factors play a critical role in its pathogenesis. Here we report two *de novo* mutations in the casein kinase 1 isoform alpha (*CSNK1A1*) gene which underlie severe epilepsy with similar clinical presentation in two patients. The identified variants are one missense mutation c.646G > C (p.Ala216Pro, Mut) in NM_001025105.3 and one deletion c.599_604delACATAC (p.His200_Ile201del, Del). *In vitro* analyses indicated that the Mut causes significant decreases in both mRNA and protein expression, while the Del demonstrated no significant impact on gene expression level. However, co-immunoprecipitation studies have shown that both mutations lead to reduced interactions between CSNK1A1 and β-catenin, resulting in excessive intracellular β-catenin and aberrant expression of several downstream genes. Compared with the wild type (WT), the EdU positive rates in cells transfected with Mut plasmid or Del plasmid were both elevated. Wnt/β-catenin signaling pathway is crucial to neurogenesis. An abnormal rise in β-catenin level has been utilized to generate genetic models for ISS. Our results not only elucidate the role of a novel candidate gene *CSNK1A1* in the pathology of ISS, but also provide further evidence for the findings that mediating Wnt/β-catenin signaling is a potential mechanism causing ISS.

## Introduction

Infantile spasms syndrome (ISS), also referred to as West Syndrome, is a common epileptic encephalopathy of infancy, characterized by uncontrollable seizures, severe electroencephalogram abnormalities, and delayed cognitive and behavioral development. It is estimated that 0.249 cases occur in 1000 live births [1]. The disease can be fatal, and it has a prevalence of approximately one in every 10 000 children at the age of 10. The onset of ISS peak between age 3 and 12 months [2]. ISS is one of the most refractory epileptic syndromes, mostly due to its high etiological heterogeneity [3]. In the pathogenesis of ISS, factors such as synaptic abnormalities, nerve growth factors, hypothalamic–pituitary–adrenal axis, and inflammation might play an important role in the development of ISS [4]. It has been reported that ISS may be associated with structural lesions, suggesting that genetic is one of the underlying etiologies [2].

Whole exome sequencing (WES) had been proven effective in the discovery of *de novo* variants underlying the complicated etiologies of epilepsy, which contributed to the development of relevant molecular diagnoses as well as new treatment targets [5, 6]. Previous studies had identified multiple mutations in ISS risk genes linked to the canonical Wnt signaling pathway [7–9]. Interestingly, an excessive β-catenin level was observed in APC conditional knock-out mice which demonstrated various salient features of human ISS, suggesting that Wnt/β-catenin signaling pathway may play a central role in the etiology of multiple ISS-inducing genetic abnormalities [10]. However, how the aberrant activation of Wnt/β-catenin signaling pathway induces ISS is still unclear.

Wnt/β-catenin signaling pathway plays an important role in neurogenesis [11–13]. *In vivo* studies had shown that this canonical Wnt signaling pathway promoted self-renewal of neural progenitor cells (NPCs) while suppressing NPC differentiation [14]. The absence of Wnt/β-catenin signaling pathway caused the premature activation of a corticogenesis transcription cascade [15]. Encoded by the *CTNNB1* gene, β-catenin is a key component of the canonical Wnt signaling pathway where it forms a degradation complex with AXIN1, AXIN2, APC, CSNK1A1 and GSK3B that promotes phosphorylation and ubiquitination of and its subsequent degradation by the proteasome [16–20]. In the presence of Wnt ligand, β-catenin accumulates in the nucleus and binds to proteins from TCF/LEF family, activating Wnt responsive genes. In this way, the expression of the responsive genes of Wnt signaling pathway is regulated by the intracellular pool of rapidly turned over β-catenin molecules.

Casein kinase 1 alpha 1, also called CK1α, is a serine/threonine kinase encoded by *CSNK1A1* gene that participates in many cellular processes, including growth and proliferation via the β catenin and Wnt signaling pathway, apoptosis, and DNA damage responses. CSNK1A1 is crucial for the sufficient phosphorylation of β-catenin by GSK3 [21]. Consequently, mutations in the gene *CSNK1A1* is predicted to affect the concentration of intracellular β-catenin as well as the expression level of Wnt targeted genes. Previous studies showed that CSNK1A1 participates in mitosis and regulates chromosome segregation in mammalian cells [22]. CSNK1A1 enables the regulation of chromosome congression and separation during mouse oocyte meiotic maturation, so *Csnk1a1* knockdown or inhibition of its activity in early embryos could lead to abnormal blastomere mitosis, thereby blocking early embryonic development. Recent studies have shown that CK1 family members are involved in many signaling pathways related to embryonic development. Although previous studies demonstrated the potential roles of CSNK1A1 in embryonic development, its roles in ISS remain elusive.

Here we elucidate the the molecular mechanism of how the *de novo* mutations in *CSNK1A1* gene found in two unrelated individuals with ISS influence Wnt signaling pathway. Recently in a systematic family-based study of ISS using WES, we identified two novel variants, c.646G > C (p.Ala216Pro) and c.599(exon6)_c.604(exon6)delACATAC (p.His200_Ile201del), within the *CSNK1A1* gene. There were no previous studies reporting correlation between *CSNK1A1* and ISS according to the gnomAD database. *CSNK1A1* encodes a serine/threonine kinase that phosphorylates β-catenin at Ser-45 [21]. The mutation sites of both variants are within the kinase domain of CSNK1A1, and they were therefore expected to show loss-of-function effects, which are speculated to be pathogenic.

## Materials and methods

### Patients and genetic analysis

The two patients were recruited from Hunan Provincial Maternal and Child Health Care Hospital. The parents of both patients were included for genetics analysis and provided informed consent. This study was approved by the Ethics Committee of Maternal and Child Health Hospital of Hunan Province (2020-S003).

For whole-exome sequencing, whole blood samples were collected with EDTA Blood Collection Tubes (Becton, Dickinson and Company, NJ, USA). DNA was extracted using FlexiGene DNA kits (Fuji, Tokyo, Japan) following the standard procedures. DNA libraries were prepared according to the instructions from Illumina (Illumina, San Diego, CA). Whole-exome enrichment was performed using the TruSeq Exome Enrichment Kit (Illumina, San Diego, CA) using the recommended protocol. Captured DNA libraries were sequenced with HiSeq2500 (Illumina, San Diego, CA, USA).

We firstly used cutadapt(v1.15) to trim adaptor sequences at the tail of sequencing reads, and then aligned sequencing reads to human reference genome (UCSC hg19) with BWA (v0.7.15). Duplicated reads were marked by Picard (v2.4.1). Qualimap (v2.2.1) was used to calculate base quality metrics, genome mapping rate, and the coverage of targeted regions. Base quality score recalibration, indel realignment and variants (SNVs & InDels) calling were performed following the best practice protocol of the Genome Analysis Toolkit (GATK, v3.8). Variant filtering was done by a finely tuned in house script. Pass-filter variants were annotated using Pubvar variant annotation engine (www.pubvar.com) and VEP (release 88). In genetic analysis, we separately identified variants that fit the dominant and recessive inheritance models. Variants met anyone of the following criteria were excluded from genetic analysis: maximum population frequency was large than 0.01, genotype confidence was low, or predicted as benign by SIFT and PolyPhen 2. The pathogenic evidences of candidate disease-causing variants were scored by InterVar (1.0.8) according to ACMG guidelines [7]. All the above analysis was performed on Seqmax (www.seqmax.com). The quality control metrics for WES of two families and genetics variants were shown in Supplementary Table 1.

### Protein structure Modeling

The tertiary structure of wild-type and mutated CSNK1A1 were built by homology-modelling using the online server SWISS-MODEL. Only one model was constructed for each session and the template chosen was 6gzd.1.A (PDB 6GZD). The effect of the variants p.Ala216Pro and p.His200_Ile201del were predicted by software PyMOL (version 2.5.2).

### Plasmid construction and mutagenesis

The coding sequence of CSNK1A1 transcript NM_001025105 was PCR amplified from cDNA using PrimeSTAR^®^ GXL Premix (Takara, Dalian, China). The N-terminal EGFP tagged vector pEGFP-C3 and the PCR amplified DNA fragments were cleaved using restriction Hind III and BamH1 with 1xK Buffer (Takara, Dalian, China). Solution I (DNA Ligation Kit, Takara) was used for ligation and the process lasted for 2 h at 16°C. Mutated plasmids were constructed with the Hieff Mut™ Site-Directed Mutagenesis Kit (YEASEN, Shanghai, China). Primers used for PCR amplification and mutagenesis are shown below, PCR forward primer: 5’-CCCAAGCTTATGGCGAGTAGCAGC-3′, PCR reverse primer: 5’-CGCGGATCCGAAACCTTTCATGT-3′, *CSNK1A1*_Mut forward primer: 5’-GCCCGATATCCTAGCATCAATGCACATCT-3′, *CSNK1A1*_Mut reverse primer: 5’-TGATGCTAGGATATCGGGCAGTGCCAGTG-3′, *CSNK1A1*_Del forward primer: 5′- CAACCATACAGAGAAGATAAAAACCTCACTGGCAC-3′, CSNK1A1_Del reverse primer: 5′- CTGTATGGTTGCCTTGTCCTGTTGTCTCTG-3′.

### Cell culture and plasmid transfection

Human embryonic kidney cells (HEK)-293 T cells were cultured in Dulbecco’s Modified Eagle’s Medium (DMEM) (HyClone, Logan, UT, USA) supplemented with 10% fetal bovine serum (FBS) (Gibco, USA) at 37°C in a humidified 5% CO_2_ atmosphere incubator. Transient transfection was performed by FuGENE^®^ HD reagent (Promega, United States) following the manufacturer’s recommendations. The cells were plated a day before transfection at a density of 5 × 10^5^ cells per well of a 6-well plate. After 24 h, plasmid DNA was added at 3.3 μg/well diluted with 155 μl Opti-MEM (Gibco, United States) and mixed with 9.9 μl of FuGENE^®^.

### Immunoblot analysis of wild type and mutant CSNK1A1

HEK-293 T cells were collected and lysed with RIPA lysis buffer (Beyotime Biotechnology, Shanghai, China). Total cell lysates were mixed with SDS-PAGE Sample Loading Buffer (Beyotime Biotechnology, Shanghai, China) and boiled for 10 min. Subsequently, 10% polyacrylamide gels were used according to recommended protocols and proteins were then transferred onto PVDF membranes (GE Healthcare, Solingen, Germany). After being blocked with 5% skim milk, the PVDF membranes were incubated overnight with primary antibodies at 4°C, followed by HRP-conjugated secondary antibodies at room temperature for 1 h (Jackson, Lansing, MI, USA; 1:5000). Blots were formed using the ECL reagent (Share-bio, Shanghai, China). After determining optimal working conditions, all experiments were conducted in triplicate. Primary antibodies used in this study were anti-GFP (Abcam, Cambridge, UK; 1:1000) and β-Catenin (D10A8) XP® Rabbit mAb (Cell Signaling Technology, Boston, USA).

### Coimmunoprecipitation

HEK-293 T cells were transfected with EGFP-tagged *CSNK1A1* and Flag-*CTNNB1*plasmids. After 48 h co-transfection, cells were lysed with a NP-40 buffer (Beyotime Biotechnology, Shanghai, China). After protein quantification, the whole-cell lysates were immunoprecipitated with anti-EGFP antibody overnight at 4°C where 500 μg protein was immunoprecipitated with 5 μg antibody. Subsequently, each IP was bound to A/G agarose beads for 4 h at 4°C (Santa Cruz Biotechnology, Dalla, TX, US). Then the beads were rinsed with RIPA buffer five times at 4°C. After final wash, aspirate supernatant and resuspend pellet with SDS-PAGE buffer. Then boil the resuspension followed by Western blot analysis. In the Western blot assay, the whole-cell lysate was classified into an input group as the positive control.

### 5-Ethynyl-2′-deoxyuridine assay

For 5-ethynyl-2′-deoxyuridine (EdU) analysis, the cells were collected after transfection. An EdU assay was performed using a BeyoClick^™^ EdU-594 Cell Proliferation Kit (Beyotime) following the manufacturer’s instructions. Images were taken by an upright fluorescence microscope (Axio Imager M2) and analyzed using software ImageJ (version 1.8.0.112). EdU-positive cells were stained red and calculated as (EdU add-in cells/DAPI stained cells) × 100%.

### qPCR

Total RNA from cultured cells was extracted with TRIzol reagent (#DP424, Tiangen biotech) and complementary DNAs (cDNAs) were synthesized using One-Step gDNA Removal and cDNA Synthesis SuperMix kit (#AT311, Transgen biotech) according to the manufacturer’s instruction. qPCR reactions were performed on an Applied Biosystems using Top Green qPCR SuperMix kit (#AQ131, Transgen biotech) and finally analyzed using LightCycler^®^ 480 Instrument II (Roche). The following primers were used as shown in the Supplementary Table 2, and *GAPDH* was used for internal control.

### Statistical analysis

Normal distribution was tested using the Kolmogorov–Smirnov test and variance was compared. All statistical analyses in this study were performed by using the Graphpad Prism 8 for macOS (version 8.4.3). Unless otherwise stated, the independent t-test evaluates whether the means for two independent groups are statistically different from each other. Continuous variables were represented as mean ± SEM. Differences were considered statistically significant when the *P* value was < 0.05.

## Results

### Clinical presentation of affected individuals

**Figure 1:**
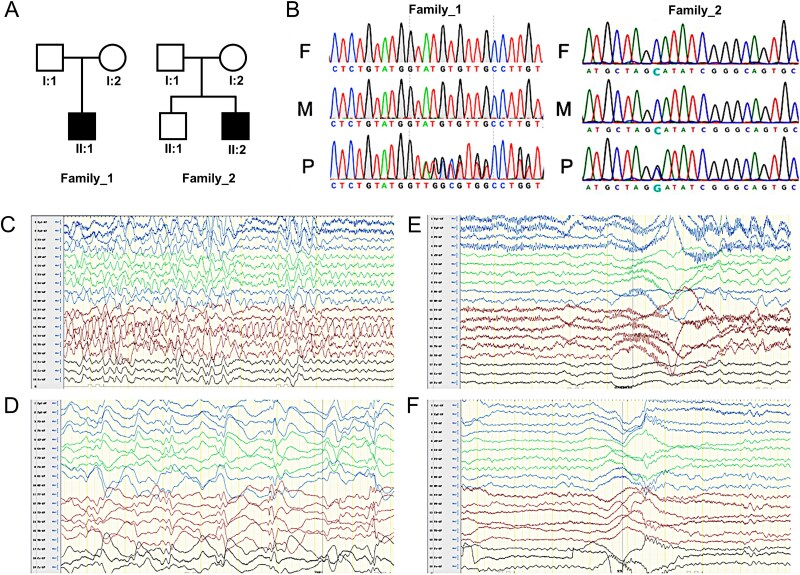
Genetic variants and EEG detection in ISS-diagnosed patients. (A) Two novel variants detected in two families with ISS- diagnosed siblings. The black squares indicated the proposita in two families. (B) Confirmation of two *CSNK1A1* variants in the patients and their parents by sanger sequencing. F: Father; M: Mother; P: Proposita. (C) and (D) EEG of patient 1 revealed intermittent partial hypsarrhythmia and clusters of spastic seizures (obtained at the age of 3 months). (E) and (F) EEG of patient 2 showed widespread spike slow waves accompany with partial hypsarrhythmia and spams at 8-month-old.

First, we performed the systematic trios-based WES in two independent ISS families and identified two de novo variants in exon 6 of *CSNK1A1*. A heterozygous missense mutation NM_001025105.3: c.646G > C(p.Ala216Pro, Mut) and a deletion variant NM_001025105.3: c.599_c.604delACATAC (p.His200_Ile201del, Del) were found in patient 1 and 2 respectively. Both variants were confirmed by Sanger sequencing (Fig. 1B).

Patient one was the first child of a non-consanguineous healthy couple of Chinese origin (Fig. 1A). He was delivered vaginally at 37 weeks and had a newborn aspiration pneumonia at birth due to amniotic fluid choking with an Apgar score of 5. At birth, he had a head circumference of 35 cm (50^th^ percentile), weight of 3 kg (50^th^ percentile), and height of 50 cm (50^th^ percentile). The child’s chest wall was concave, suggesting congenital funnel chest. Fundus screening suggested congenital glaucoma. Cardiac ultrasound suggested congenital heart disease, manifested by cardiac ventricular septal defect, unclosed ductus arteriosus, and unclosed oval hole. At 1 month of age, he was found to have feeding difficulties, growth retardation and poor response, and at 2 months, he was found to have a peculiar facial feature, including a small jaw, short nasal bridge and sparse yellow hair. At 3 months, the child developed nodding spasms, EEG showed abnormal peak rhythm (Fig. 1C and D), and cranial magnetic resonance showed cerebral hypoplasia; the spasms were slightly controlled by treatment with adrenocorticotropic hormone, sodium valproate, topiramate, and vinyl chloride. At 10 months old, the child was still unable to hold his head upright, roll over, sit alone, grasp, and had a head circumference of 45 cm (50th percentile), weight of 6 kg (<−3 SD), and height of 70 cm (−1 SD). The child died of severe malnutrition at 1 year of age. His mother had a normal pregnancy with no history of infection, toxic exposure, or exposure to radioactive materials during pregnancy. No family history of neurological disease was reported by the parents.

Patient two is a currently 9-year-old boy born to non-consanguineous Chinese parents with a normal developing brother (Fig. 1A). The mother had a normal pregnancy and at 39 weeks the patient was delivered vaginally with no birth injuries and no history of hypoxic asphyxia at birth. At birth, his head circumference, height and weight were in the 50th percentile. At 2 months of age, the patient was found to have a short, collapsed nasal bridge and poor eye spacing, and at 6 months of age, the child was found to be developmentally delayed, showing inability to roll over and sit alone, and poor response to sound chasing. The epileptic seizures were controlled but occurred once every 3–6 months. The seizures were controlled, with seizures occurring once every 3–6 months. At 8 months EEG demonstrated aberrant peak rhythm (Fig. 1E and F). He could barely walk on his feet until he was about 1.5 years old, and at 3 years old, he was found to be prone to crying, irritability, difficulty concentrating, and small movements. During a systematic evaluation at age 8, he was diagnosed with mental retardation, attention deficit and hyperactivity disorder after scoring 59 on the verbal intelligence quotient (VIQ), 62 on the performance intelligence quotient (PIQ), 56 on the full-scale intelligence quotient (FIQ), 84 on the Autism Behavior Rating Scale, and 26 on the Kirschner Autism Behavior Inventory. He currently has learning difficulties, can only perform some simple calculations, is hyperactive, has average emotional control, and performs poorly in social activities. A negative family history of neurological diseases was reported by the parents.

### *In silico* predictions of *de novo* variants and effects on gene expression

**Figure 2:**
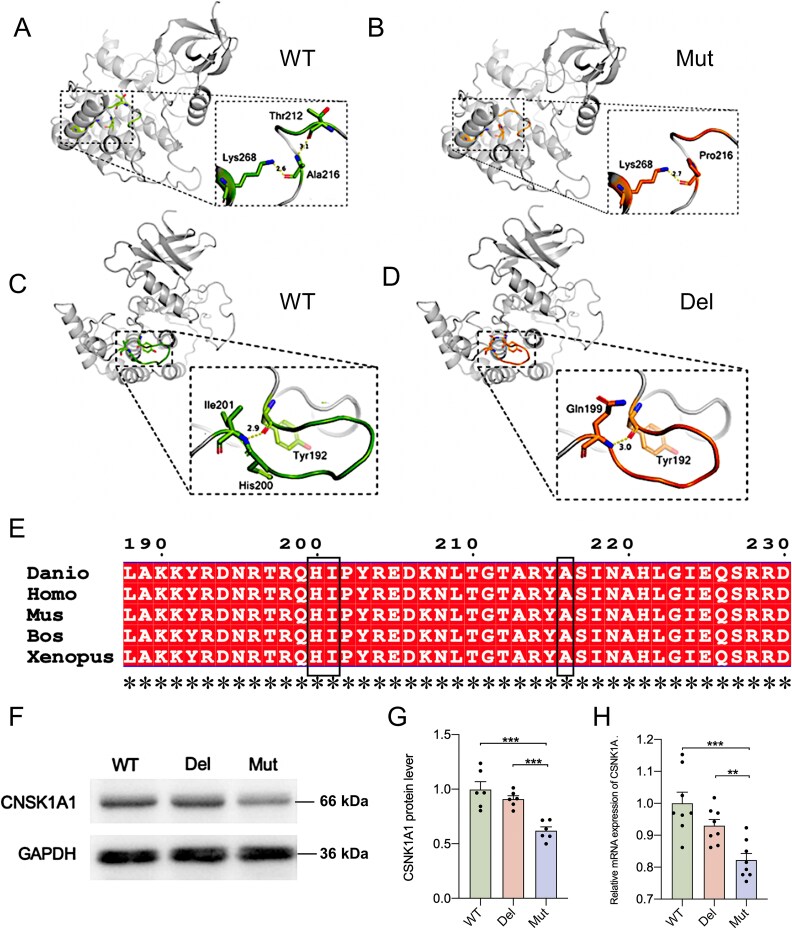
Genetic variants detected *in silico* prediction and gene expression. (A) and (B) structural analysis of the CSNK1A1WT and CSNK1A1p.Ala216Pro (Mut); (C) and (D) structural analysis of the CSNK1A1WT and CSNK1A1p.His200_Ile201del (Del). F: Father; M: Mother; P: Proband. *In vitro* functional study of p.Ala216Pro and p.His200_Ile201del. (E) Conservation of p.Ala216 and p.His200_Ile201 in mammals. (F) and (G) the CSNK1A1 protein expression was down-regulated in Mut in comparison to WT (*n* = 6, ^**^P < 0.01). (H) the mRNA expression of *CSNK1A1* was decreased in Mut compared to WT according to the result of qPCR (*n* = 8, ^**^P < 0.01, ^***^P < 0.001).

Then we performed *in silico* predictions to investigate the deleteriousness and possible function of the mutations. The missense mutation p.Ala216Pro was predicted to be damaging by Polyphen2 and SIFT. Protein structure and noncovalent interaction analysis were conducted with WT CSNK1A1 and the variants. A polar interaction between the side chains of Ala216 and Thr212 was found, which was abolished by the p.Ala216Pro substitution (Fig. 2A and B). A loop was shortened by 2 amino acid residues due to the deletion p.His200_Ile201del, but the interaction between the main chain of Tyr192 and Ile 201 was replaced by a main-chain interaction between Tyr192 and Gln199 (Fig. 2C and D). Based on the alignment of amino acid sequence among vertebrates, we found that the residue p.Ala216 and p.His200_Ile201 (shown in the red frame) in CSNK1A1 is highly conserved among these species (Fig. 2E).

Next, *in vitro* experiments were carried out to study the functions of the variants. The results of qPCR and Western blot assays demonstrated that Mut remarkably reduced *CSNK1A1* gene expression on both mRNA and protein level. However, no significant difference was observed between WT and Del despite the slight decreasing trend (Fig. 2F–H).

### Effects on protein–protein interaction and β-catenin expression

**Figure 3:**
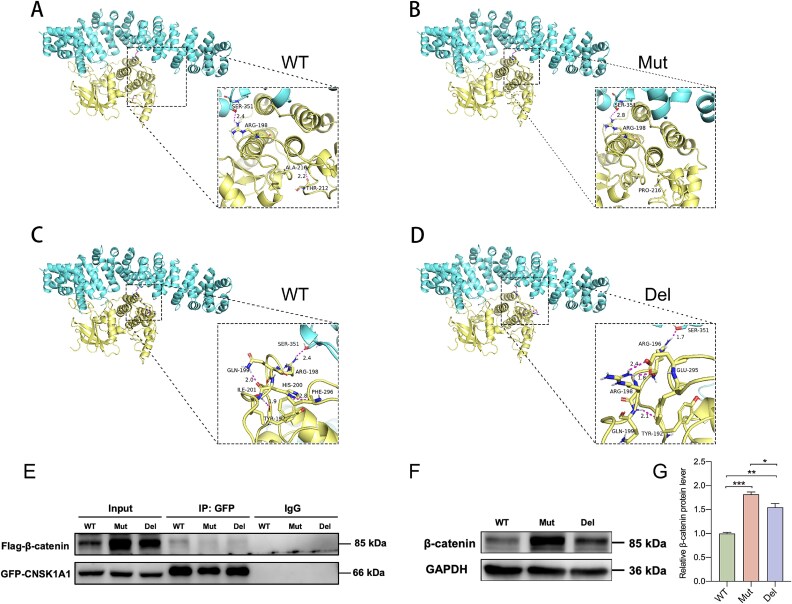
*In silico* prediction and Co IP detection of CSNK1A1-β-catenin. (A) and (B) *in silico* protein–protein interaction analysis of β-catenin and CSNK1A1 WT or CSNK1A1 p.Ala216Pro (Mut); (C) and (D) *in silico* prediction analysis of β-catenin and CSNK1A1 WT or CSNK1A1 p.His200_Ile201del (Del). (E) the pulled-down β-catenin was significantly less in Mut and Del than in WT as demonstrated by Co-IP analysis. (F) and (G) increased intracellular level of β-catenin was induced by both Mut and Del (^*^P < 0.05, ^**^P < 0.01, ^***^P < 0.001).

Protein structure and noncovalent interaction analysis were conducted with WT CSNK1A1 and the variants. The interaction between the CSNK1A1 and β-catenin is mediated by hydrophobic and polar interactions. The binding interface of CSNK1A1-β-catenin can be subdivided into many binding sites/regions. In detail, A polar interaction between β-catenin Ser351 and CSNK1A1 Arg198 was found, which was influenced by the p.Ala216Pro substitution (Fig. 3A and B). The loop included the CSNK1A1 p.Arg198 was shortened by two amino acid residues due to the deletion p.His200_Ile201del. Although the deletion p.His200_Ile201del resulted in the polar interaction disappear between the β-catenin Ser351 and CSNK1A1 Arg198, the polar interaction was reconstructed coincidentally between β-catenin Ser351 and CSNK1A1 Arg196 (Fig. 3C and D). Although the two variants didn’t abolish the polar interaction between β-catenin Ser351 and CSNK1A1 Arg198/Arg196, they do indeed affect the length of the chemical bonds between amino acids. The above results indicate that the variants might lead to conformational changes in CSNK1A1, which then disrupts the interaction between β-catenin and CSNK1A1.

We then tested whether CSNK1A1 substrate binding was inhibited by the variants. The results of immunoprecipitation indicated that, in both Mut and Del, the interaction between CSNK1A1 and β-catenin was considerably decreased (Fig. 3E). Moreover, the intracellular level of β-catenin was strongly enhanced in both variants (Fig. 3F and G), suggesting that the β-catenin degradation complex failed to control the concentration of β-catenin when the Wnt/β-catenin pathway was deactivated. Noticeably, more β-catenin remained in Mut cells than in Del cells.

### Effects on cell proliferation and canonical Wnt signaling pathway gene expression

**Figure 4:**
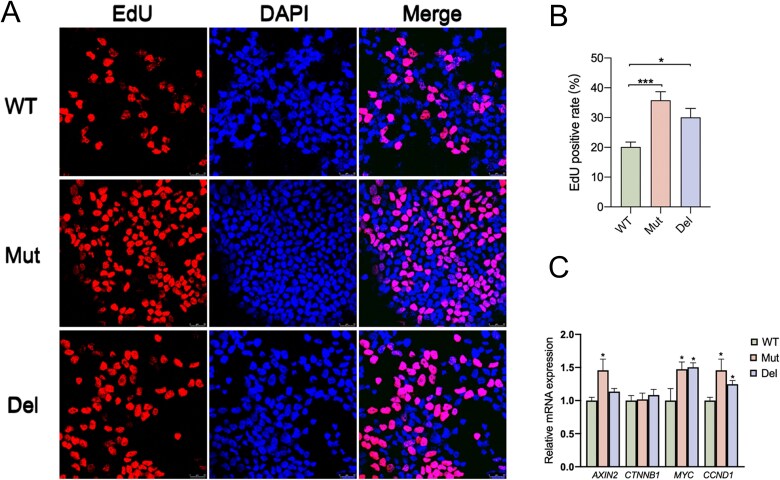
Variants promotes proliferation of HEK-293 T cells and Wnt pathway gene expression. (A) and (B) detection of EdU add-in cells 48 h after transfection. Nuclei were counterstained with DAPI. EdU-positive cells were stained red and calculated as (EdU add-in cells/DAPI stained cells) × 100%. (C) the mRNA expression of *MYC* and *CCND1* were increased in both Mut and Del. Only Mut induced increase in AXIN2 expression and both variants did not affect the expression of β-catenin. (^*^P < 0.05, ^***^P < 0.001).

Since β-catenin plays a critical role in Wnt signaling, entering the nucleus and binding to transcription factors, we inferred that the rise in β-catenin would aberrantly activate the expression of Wnt targeted genes. Therefore, we performed qPCR on genes *AXIN2*, *MYC* and *CCND1* and observed significantly higher expression levels of *MYC* and *CCND1* in both Mut and Del. However, among the two variants, only Mut resulted in higher level of *AXIN2* transcription (Fig. 4C).

Given that *MYC* and *CCND1* contribute to accelerating cell cycle progression, we then tested whether cell proliferation was facilitated by the abnormally mediated gene expression. HEK-293 T cells were transfected with WT, Mut or Del plasmid respectively, and were examined by an EdU assay. Compared with the WT, the EdU positive rates (percentage of cells that had undergone cell division) in cells transfected with Mut and Del were elevated (Fig. 4A and B).

## Discussion

In this study, we identified two *de novo* ISS-inducing variants of *CSNK1A1* c.646G > C, c.599_c.604delACATAC in exon 6 based on WES. To the best of our knowledge, our study is the first one to report that mutations in this gene could cause ISS.

The mutation variant c.646G > C caused decreased mRNA and protein expression while the deletion variant c.599_c.604delACATAC did not affect gene expression on a significant level. As indicated by Co-IP results, the two variants inhibited the binding of β-catenin to CSNK1A1 with the missense mutation variant showed more intensive disturbance. Both variants located within the kinase domain of CSNK1A1 that could influence the protein substrate binding process [23]. Through *in silico* predictions we found that the p.Ala216Pro substitution was influenced the polar interaction between β-catenin and CSNK1A1, which was critical for the effective phosphorylation at Ser45 [21]. Spatial resistance introduced by the substitution of Proline for Alanine would potentially change the protein structure. The other variant, p.His200_Ile201del, was on a flexible loop. It did not affect the orientation of the loop, but shortened it by two residues. The distinction in the structural effects on CSNK1A1 might explain for the difference between CSNK1A1 p.Ala216Pro and CSNK1A1 p.His200_Ile201del in β-catenin binding ability. As for the first patient exhibiting more severe EEG abnormalities and other phenotypic features. There could be several reasons for this discrepancy between the clinical and molecular severity. Each patient has a unique genetic background. Other genes in the genome can act as modifiers, either enhancing or dampening the effects of the *CSNK1A1* mutations. There could also be genetic modifiers in genes related to neural development or synaptic plasticity may influence how the *CSNK1A1* mutation manifests clinically, while not directly affecting the measured molecular parameters in the in-vitro assays.

β-catenin is a key downstream component of the canonical Wnt signaling pathway. Canonical Wnt signaling pathway was found to tip the balance between NPC self-renewal and NPC differentiation towards the former [14, 15]. Multiple studies had identified ISS risk genes associated with β-catenin and Wnt signaling pathways including *S-SCAM/Magi2*, *TSC1/2*, *FoxG1*, *ARX* and *APC* [9, 10, 24–30]. Similar to CSNK1A1, APC serves as a negative regulator for Wnt signaling pathway by promoting the rapid degradation of β-catenin [31, 32]. Previous studies had found that APC conditional knockout in mouse could lead to the presentation of multiple clinical features of human ISS [10]. Given their cooperative role in the function of β-catenin degradation, we suspected that the regulation of β-catenin played a key role in the etiology of ISS.

CSNK1A1 is essential in Wnt/β-catenin signaling pathway where it phosphorylates β-catenin at Ser45, preparing β-catenin for sufficient phosphorylation by GSK3 which eventually leads to its degradation. Our study demonstrated that variant p.Ala216Pro induced severer accumulation of β-catenin in cells than variant p.His200_Ile201del did, which could be interpreted as stronger interference with canonical Wnt signaling pathway. This could illustrate why patient one suffered more serious clinical outcomes to some extent. However, from either mRNA expression level or EdU positive rate, we concluded that the boosting effect on cell proliferation from both variants were similar. A possible explanation is that the underlying mechanism of how disruption in Wnt signaling pathway causes ISS involves more responsive genes than those mediating cell cycle. That is to say, the expression of Wnt targeted genes are affected in various levels according to the nucleic concentration of β-catenin, which eventually leads to distinct phenotypes of ISS. Interestingly, in our study we noticed that only p.Ala216Pro enhanced the expression of *AXIN2* while p.His200_Ile201del showed no major effect. Since AXIN2 is a critical component of β-catenin degradation complex, this result could support the proposed explanation by demonstrating the correlation between higher β-catenin level, stronger feedback regulation and severer ISS phenotypes.

In conclusion, our findings confirmed that, with variable severity, both de novo heterozygous variants of *CSNK1A1* underlay infantile spasms syndrome. The *in vitro* studies elucidated that variants in *CSNK1A1* resulted in ISS by raising intracellular β-catenin level and disturbing the Wnt/β-catenin signaling pathway, which supported previous studies on the roles of β-catenin-dependent pathway malfunction in the molecular etiology of ISS. Further *in vivo* studies are required to illuminate how interference to Wnt/β-catenin signaling pathway contributed to the pathogenesis of ISS. It will also be interesting to investigate whether correction in activation of Wnt/β-catenin signaling pathway in *CSNK1A1* variants will amend the aberrant cell proliferation. More insights into the crucial role of Wnt/β-catenin signaling pathway in cortical development and neural circuit function will facilitate improvements in therapeutic interventions with potential targets.

In summary, our results not only elucidate the role of a novel candidate gene *CSNK1A1* in the pathology of ISS, but also provide further evidence for the findings that mediating Wnt/β-catenin signaling is a potential mechanism causing ISS. Despite our efforts to establish a link between *CSNK1A1* gene variants and ISS, this study has several limitations. Firstly, the sample size, although carefully selected, is relatively small. This may limit the statistical power to detect subtle associations between the gene variants and IS, and the results may not be fully representative of the entire ISS patient population. *In vitro* experiments, while providing valuable insights into the cellular and molecular mechanisms, cannot fully recapitulate the complex physiological environment of the human body. Finally, our understanding of the underlying mechanisms through which *CSNK1A1* gene variants lead to ISS is still incomplete. Although we have proposed a preliminary molecular mechanism, there are likely other genes and pathways that interact with CSNK1A1 and contribute to the development of ISS. Further research is required to comprehensively elucidate the complex network of genetic and molecular factors involved in the pathogenesis of ISS.

## Supplementary Material

Supplementary_table_ddaf030
