# Supplementary material for: The role of CSNK1A1 and its de novo mutations in infantile spasms syndrome
Source: Hum Mol Genet. 2025 Mar 28;34(10):905–13. doi: 10.1093/hmg/ddaf030 (PMC12056308; doi:10.1093/hmg/ddaf030)
Supplement: Supplementary_table_ddaf030 [file supplementary_table_ddaf030.docx]

Table 1. Genetic variants not directly linked to the clinical phenotype of the ISS patients

| **Gene and transcript** | **Location** | **Nucleotide and amino acid changes** | **Genotype** | **Population frequency** | **Pathogenicity classification** | **Disease/Phenotype [Inheritance pattern]** | **Origin** |
| --- | --- | --- | --- | --- | --- | --- | --- |
| ACTN1  NM_001130004.1 | 14:69376712 | c.472_475delACAG  p.Thr158ProfsTer13 | Het | - | LP | Platelet-type bleeding disorder 15; BDPLT15[AD] | De novo |
| USH2A  NM_206933.2 | 1:216419934 | c.2802T>G  p.Cys934Trp | Het | 0.004 | P | 1. Usher syndrome type 2A; USH2A[AR]  2. Retinitis pigmentosa 39; RP39[-] | Paternal |
| ROBO3  NM_022370.3 | 11:124740059 | c.767-2A>G | Het | - | P | Familial horizontal gaze palsy with progressive scoliosis; HGPPS[AR] | Paternal |
| ROBO3  NM_022370.3 | 11:124740060 | c.767-1G>C | Het | - | P | Familial horizontal gaze palsy with progressive scoliosis; HGPPS[AR] | Paternal |
| SOHLH1  NM_001012415.2 | 9:138589474 | c.346-1G>A | Het | 0.0106 | P | 1. Spermatogenic failure 32; SPGF32[AD]  2. Ovarian dysgenesis 5; ODG5[AR] | Paternal |
| FBN1  NM_000138.4 | 15:48719886 | c.7082C>T  p.Ser2361Leu | Het | 0.00000815 | LP | 1. Marfan syndrome; MFS[AD]  2. Mass syndrome [-]  3. Geleophysic dysplasia 2; GPHYSD2[AD]  4. Stiff skin syndrome; SSK[AD]  5. Marfan lipodystrophy syndrome; MFLS[AD]  6. Weill-Marchesani syndrome type 2; WMS2[AD]  7. Acromicric dysplasia; ACMICD[AD]  8. Autosomal dominant simple ectopia lentis type 1; ECTOL1[AD] | Maternal |
| SLCO1B1  NM_006446.4 | 12:21370120 | c.1568_1571dupAATG  p.Cys524Ter | Het | 0.000058 | LP | Rotor type hyperbilirubinemia; HBLRR[DR] | Paternal |
| DISC1  NM_018662.2 | 1:231954090 | c.1808C>T  p.Thr603Met | Het | 0.00029 | LP | Schizophrenia 9; SCZD9[-] | Maternal |
| TTC37  NM_014639.3 | 5:94857860 | c.1909A>G  p.Lys637Glu | Het | 0.00324 | LP | Tricho-hepato-enteric syndrome 1; THES1[AR] | Paternal |
| PCDH15  NM_033056.3 | 10:55663053 | c.3451G>A  p.Gly1151Arg | Het | 0.0198 | LP | 1. Autosomal recessive nonsyndromic hearing loss 23; DFNB23[AR]  2. Usher syndrome type 1D; USH1D[AR,DR]  3. Usher syndrome type 1F; USH1F[AR] | Maternal |
| WDR62  NM_001083961.1 | 19:36549693 | c.189G>T  p.Glu63Asp | Het | 0.00887 | P | Primary autosomal recessive microcephaly 2, with or without cortical malformations; MCPH2[AR] | Maternal |
| CFTR  NM_000492.3 | 7:117267766 | c.3659C>T  p.Thr1220Ile | Het | 0.00186 | P | 1. Congenital bilateral absence of the vas deferens; CBAVD[AR]  2. Bronchiectasis with or without elevated sweat chloride 1; BESC1[AD]  3. Hereditary pancreatitis; PCTT[AD]  4. Cystic fibrosis; CF[AR] | Maternal |
| TSC1  NM_000368.4 | 9:135781505 | c.1460C>G  p.Ser487Cys | Het | 0.00185 | LP | 1. Lymphangioleiomyomatosis; LAM[-]  2. Taylor-type focal cortical dysplasia; FCDT[-]  3. Tuberous sclerosis 1; TSC1[AD] | Paternal |

Note: The listed variants may be related to the patient's clinical phenotype, but cannot fully explain the clinical presentation. This includes the following scenarios: 1) The detected variant has uncertain clinical significance; 2) The genotype of the tested individual does not align with the genetic inheritance pattern of the disease; 3) The patient's clinical phenotype does not match the main clinical features of the disease.

Table 2. Primer Sequence for qPCR

| **Gene name** | **Primer forward (5’ to 3’)** | **Primer reverse (5’ to 3’)** |
| --- | --- | --- |
| *CSNK1A1* | ACTATTGTCGTGGGCTACG | TTGATGGTTCAGGGTCCTG |
| *CTNNB1* | CCAAGTCCTGTATGAGTGGG | GCATACTGTCCATCAATATCAGC |
| *MYC* | ATTCTCTGCTCTCCTCGAC | TTGTTCCTCCTCAGAGTCG |
| *CCND1* | GCGTACCCTGACACCAATCTC | ACTTGAAGTAAGATACGGAGGGC |
| *AXIN2* | CCTGGCTCCAGAAGATCAC | GTAAGTGACAACCAACTCACTG |
| *GAPDH* | TCAAGATCATCAGCAATGCC | CGATACCAAAGTTGTCATGGA |
